# Association between long-term ambient air pollution and incident inflammatory bowel disease across levels of adherence to a plant-based diet: a prospective cohort study in the UK Biobank

**DOI:** 10.3389/fnut.2026.1893740

**Published:** 2026-08-10

**Authors:** Lili Liu, Kequan Chen, Di Liu, Jinjian Chen, Siru Yang, Su Yao, Zhenzhang Li, Jun Yang, Nannan Chen

**Affiliations:** 1 School of Public Health, Guangzhou Medical University, Guangzhou, China; 2 Department of Gastroenterology, The First Affiliated Hospital of Guangzhou Medical University, Guangzhou, China; 3 Hospital-Acquired Infection Control Department, ShunDe Hospital of Guangzhou University of Chinese Medicine, Foshan, China; 4 Department of Pathology, Guangdong Provincial People's Hospital, Guangdong Academy of Medical Sciences, Guangzhou, China; 5 College of Mathematics and Systems Science Guangdong Polytechnic Normal University, Guangzhou, China

**Keywords:** air pollution, cohort studies, ulcerative colitis, Crohn disease, plant-based diet, inflammatory bowel diseases

## Abstract

**Background:**

Ambient air pollution is a potential environmental risk factor for inflammatory bowel disease (IBD), whereas a plant-based diet may be protective. However, whether such a dietary pattern modifies the association between long-term air pollution exposure and IBD risk remains unclear.

**Methods:**

This prospective cohort study included 165,722 participants from the UK Biobank. Annual average concentrations of ambient air pollutants were estimated using air dispersion models. The plant-based diet index (PDI) was calculated based on the intake of 17 major food groups recorded in the 24-h dietary recall. Time-dependent Cox regression was applied to examine the association between long-term air pollution exposure and IBD risk, and stratified analyses along with interaction terms were performed to evaluate potential modification by the PDI.

**Results:**

This study demonstrated a positive association between air pollution exposure and the incidence of IBD, regardless of pollutant type. Air pollution exposure was positively associated with IBD risk among participants with lower PDI, whereas the point estimates were smaller and no longer statistically significant among those with higher PDI. A similar pattern was observed for ulcerative colitis.

**Conclusion:**

Long-term exposure to ambient air pollution may be associated with an increased risk of IBD. In the higher plant-based diet group, air pollution-related IBD risk was relatively lower, providing additional epidemiological evidence into the prevention and management of IBD.

## Graphical abstract

**Figure:**
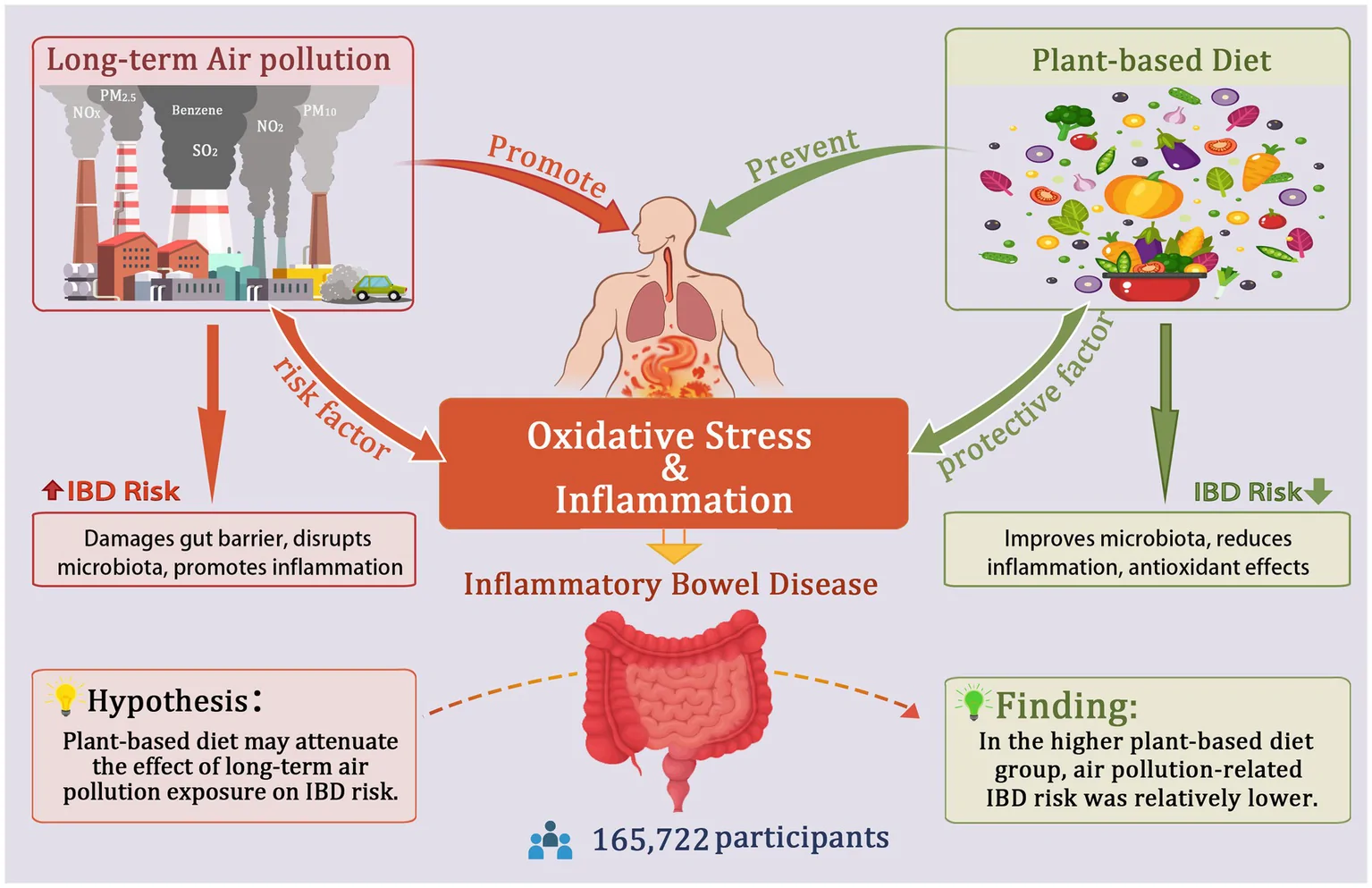

## 1 Introduction

Inflammatory bowel disease (IBD) comprises immune-mediated disorders characterized by chronic, relapsing inflammation of the gastrointestinal mucosa, primarily Crohn’s disease (CD) and ulcerative colitis (UC) (1). Its global burden has risen substantially in recent decades, with the Global Burden of Disease 2021 study reporting an 88.3% increase in new cases from 1990 to 2021 (2). As no curative therapy exists, patients typically require long-term pharmacological treatment and ongoing disease management, imposing significant personal and socioeconomic burdens (3, 4). Thus, identifying modifiable environmental and lifestyle factors is essential for IBD prevention.

The pathogenesis of IBD is complex, involving interactions between genetic susceptibility and environmental factors, with environmental exposures playing a key role in disease onset and progression (5, 6). In recent years, rapid industrialization and urbanization have led to substantial increases in global air pollution levels (7). According to the State of Global Air 2025 report from the Health Effects Institute (HEI), air pollution remains the second leading risk factor for premature mortality worldwide, surpassed only by hypertension (8). Extensive epidemiological evidence links air pollution to elevated risks of various chronic diseases, including cardiovascular and respiratory conditions, diabetes, and multiple cancers (9–12), and growing data indicate adverse effects on digestive health as well (13). A few studies have also suggested associations between air pollution exposure and accelerated disease progression or increased all-cause mortality among individuals with IBD (14–16). However, prior studies have typically examined only a narrow set of pollutants and mainly used non-prospective designs (17, 18). Large-scale prospective cohorts systematically assessing associations between multiple air pollutants and IBD incidence remain scarce.

Among lifestyle factors, dietary and nutritional intake is a key modifiable determinant of human health, and optimizing dietary intake is increasingly recognized as an important strategy for the prevention of chronic diseases (19). Evidence from randomized trials and systematic reviews suggests that dietary interventions and natural products may help improve blood pressure regulation and glycemic control (20, 21). Among various dietary patterns, plant-based diets have received growing attention owing to their protective associations with a range of chronic diseases. A comprehensive systematic review and meta-analysis suggests that greater adherence to plant-based dietary patterns is associated with reduced risks of major chronic diseases, including type 2 diabetes, cardiovascular disease, cancer, and premature mortality (22). Key components of plant-based diets, including dietary fiber, polyphenols, and unsaturated fatty acids, have anti-inflammatory and antioxidant properties, which may contribute to their protective associations with chronic inflammatory diseases such as IBD (23, 24).

Evidence indicates that diet plays a critical role in shaping the gut microenvironment and influencing the onset and progression of IBD (25). Studies have shown that high intake of processed meat and animal protein is positively associated with the incidence of IBD and adverse clinical outcomes (26, 27). In contrast, a plant-based dietary pattern may confer protective benefits by improving gut microbial balance and reducing pro-inflammatory metabolites (28), and semi-vegetarian diets have been shown to help maintain IBD remission (29). In addition, a recent multinational retrospective cohort study suggested that adherence to plant-based diet indices was associated with the risk of incident IBD and disease prognosis (30). Thus, a plant-based dietary pattern may have potential value in IBD prevention and management.

Oxidative stress and inflammatory responses are key biological pathways through which air pollution exerts harmful effects (31). Given that air pollution may elevate IBD risk by promoting inflammation, whereas a plant-based diet exerts anti-inflammatory and potential protective effects, biological interactions between these pathways are biologically plausible. However, it remains unclear whether adherence to a plant-based diet moderates the association between air pollution exposure and the risk of IBD. Using the large prospective UK Biobank cohort, this study systematically evaluated the associations between multiple air pollutants and IBD incidence, and further examined the potential modifying role of a plant-based dietary pattern.

Previous studies have mainly focused on the independent associations of dietary intake, natural compounds, or air pollution with chronic diseases (32, 33). In the context of IBD, existing studies on air pollution and IBD risk have primarily focused on single or limited environmental exposures, whereas prospective evidence simultaneously incorporating multiple air pollutants and further examining whether adherence to a plant-based diet may modify the association between long-term ambient air pollution exposure and incident IBD remains limited. Our findings provide more comprehensive epidemiological evidence from both environmental and dietary perspectives, with potential implications for understanding IBD risk and informing public health strategies.

## 2 Methods

### 2.1 Study population and exclusion criteria

**Figure 1:**
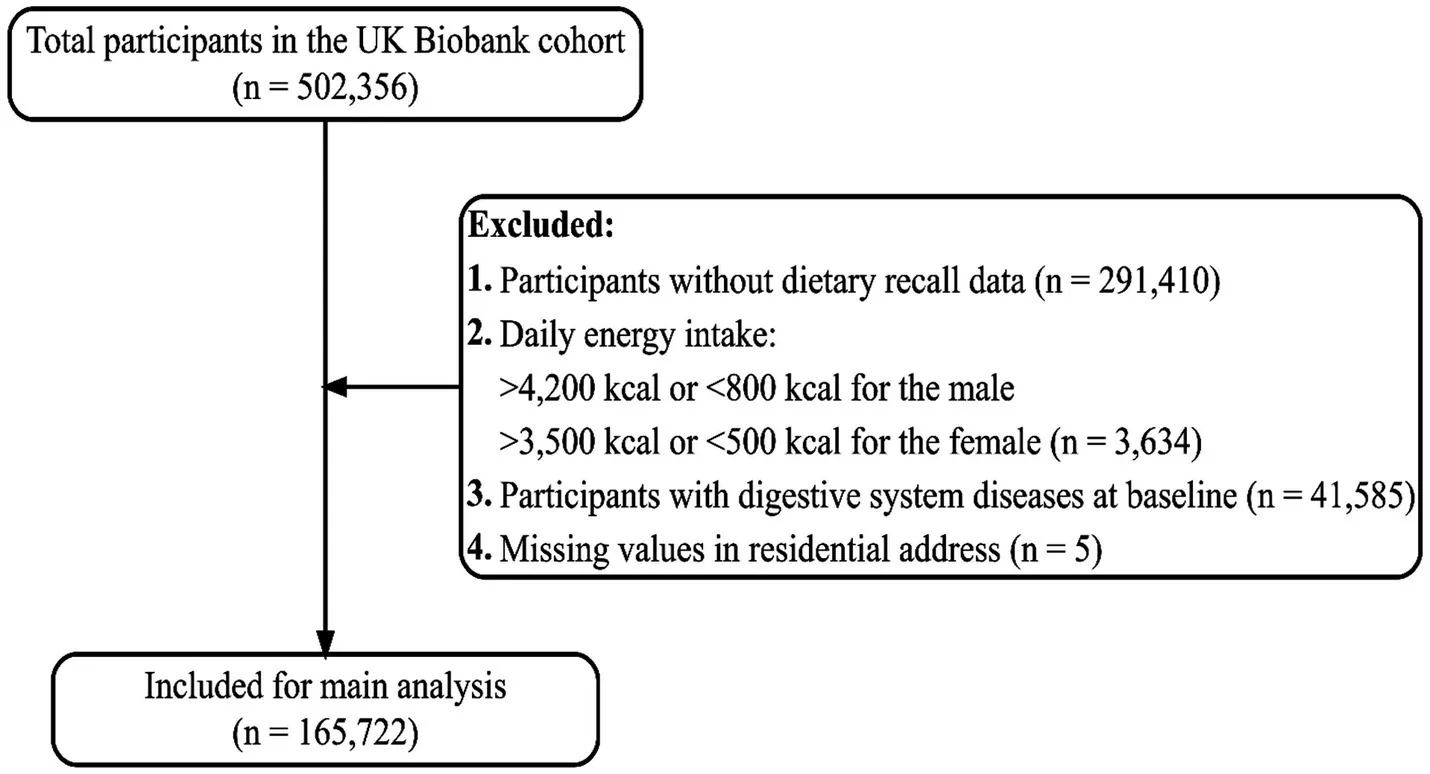
Flowchart of the participant selection.

The UK Biobank is a large-scale, population-based prospective cohort study that recruited over 500,000 participants aged 40–69 years from England, Scotland, and Wales between 2006 and 2010 (34). All participants completed baseline assessments at one of 22 assessment centers, which included touchscreen questionnaires, physical measurements, and biological sample collection (35). The baseline data included sociodemographic characteristics, lifestyle factors, and health-related information. Subsequent follow-up was conducted by linking participants’ data to electronic health records from the UK National Health Service (NHS), which allowed for long-term monitoring of health outcomes. These linked data encompassed hospital admissions, cancer registrations, and death registrations. In this study, we excluded participants who did not complete the 24-h dietary assessment; those with unreasonable energy intake (men >17,573 kJ/day or <3,347 kJ/day; women >14,644 kJ/day or <2,092 kJ/day); Participants with pre-existing digestive system diseases at baseline were excluded, with detailed disease definitions provided in Supplementary Table S1; participants with missing address information were also excluded. The final analysis included 165,722 participants (Figure 1).

### 2.2 Outcome ascertainment

The primary outcome of this study was IBD, including subtypes of CD and UC. The diagnosis of IBD and its subtypes was defined according to the International Statistical Classification of Diseases and Related Health Problems, Tenth Revision (ICD-10) codes (K50 for CD, K51 for UC, and K50–K51 for IBD). The outcome event was defined as the date of the first IBD diagnosis during follow-up. Participants were followed from the baseline assessment date until the earliest of the following events: first IBD diagnosis, death, loss to follow-up, or the end of the study period.

### 2.3 Covariates

Based on previous studies (36), potential confounders in this study included the following: sex (female, male), age (years, continuous), ethnicity (white, other), employment status (in paid employment or self-employed, retired, unemployed), educational qualifications [years of education corresponding to the International Standard Classification of Education (ISCED): 7, 10, 13, 15, 19, and 20], household income (≤£30,999, >£30,999), physical activity (based on the International Physical Activity Questionnaire [IPAQ]-derived metabolic equivalent task [MET] levels: low, <600 MET-min/week; moderate, 600–3,000 MET-min/week; high, >3,000 MET-min/week), smoking status (never, previous, current), alcohol consumption status (never, previous, current), and body mass index [BMI, categorized according to the World Health Organization (WHO) criteria: underweight (<18.5 kg/m^2^), normal weight (18.5–24.9 kg/m^2^), overweight (25.0–29.9 kg/m^2^), and obese (≥30.0 kg/m^2^)].

### 2.4 Air pollution assessment

Air pollution data used in this study were obtained from the UK Department for Environment, Food & Rural Affairs (DEFRA) through the UK-AIR platform. DEFRA employs an advanced atmospheric dispersion modelling framework to generate annual mean concentrations of multiple pollutants at a spatial resolution of 1 × 1 km. This system integrates information from the National Atmospheric Emissions Inventory (NAEI), meteorological data, and various emission sources, including road traffic, dust resuspension, and secondary inorganic aerosols. Model outputs were calibrated using data obtained from background monitoring network sites. DEFRA further validated model reliability by comparing modelled annual average pollutant concentrations with measured concentrations. Model performance was assessed using comparisons between modelled and measured annual mean air pollutant concentrations. The measured data showed good agreement with modelled estimates. Summary statistics on model performance are available in the 2020 DEFRA PCM technical report (37).

Following previously established methods (18, 36), we estimated participants’ average exposure levels during follow-up to particulate matter with aerodynamic diameter <2.5 μm (PM_2.5_), particulate matter with aerodynamic diameter <10 μm (PM_10_), nitrogen dioxide (NO_2_), nitrogen oxides (NO_x_), sulfur dioxide (SO_2_), and benzene. To fully capture the spatial heterogeneity of air pollutants and account for the influence of residential mobility on long-term exposure assessment, participants’ geospatial residential histories were linked to annual mean air pollutant concentration data provided by DEFRA using linear interpolation. This procedure assigned the corresponding annual pollutant concentration to each residential address. Subsequently, the duration of residence at each address was incorporated to time-weight pollutant concentrations across different locations, thereby deriving annually updated time-weighted air pollution exposure concentrations for each participant during follow-up.

### 2.5 Assessment of plant-based diet index

Dietary intake was assessed using repeated 24-h dietary recalls collected through the Oxford WebQ questionnaire in the UK Biobank. The Oxford WebQ is a validated web-based 24-h dietary recall instrument that records detailed information on 202 commonly consumed food items and 32 types of beverages.^1^ The UK Biobank conducted five rounds of 24-h dietary assessments using the Oxford WebQ questionnaire. The first dietary assessment was administered at the baseline assessment centre between April 2009 and September 2010. The second assessment was conducted between February 2011 and April 2011; the third between June 2011 and September 2011; the fourth between October 2011 and December 2011; and the fifth between April 2012 and June 2012. The Oxford WebQ has been validated against interviewer-administered 24-h dietary recalls conducted on the same day, with Spearman’s correlation coefficients for most nutrient intakes ranging from 0.5 to 0.9 (mean 0.6) (38). In addition, validation evidence using objective reference measures, such as urinary biomarkers and accelerometry-based estimates of energy expenditure, has shown that the measurement error properties of the Oxford WebQ were broadly comparable to those of interviewer-administered multiple-pass 24-h dietary recalls (39). Dietary data derived from the validated Oxford WebQ have also been shown to be reliable for dietary pattern assessment (40). This study included participants who completed at least one 24-h dietary recall. For individuals who completed two or more dietary assessments, the mean intake across all available records was used for the analysis to reduce within-person variation and improve the reproducibility of dietary intake estimates.

The PDI was constructed based on methods used in previous studies (41, 42). Because the UK Biobank dataset does not include information on plant oil consumption, foods were classified into 17 food groups. Overall, intakes of most food groups were categorized into quartiles, with zero intake treated as a separate category, resulting in five levels (scores 1–5). Plant-based food groups were assigned positive scores (higher intake indicating higher scores), whereas animal-based food groups were assigned reverse scores (higher intake indicating lower scores). In addition, based on previous literature (42), the animal fat group was categorized into two levels (scores 1 or 3), the nut group was classified into tertiles (scores 3–5), while the remaining food groups were scored according to quartiles. The intake of each food or beverage was calculated by multiplying the assigned portion size by the reported amount consumed, thereby generating a standardized and quantified measure of intake (43). Finally, the overall PDI score was obtained by summing the scores of the 17 food groups. Participants were then categorized into tertiles of PDI, representing low, moderate, and high adherence to a plant-based diet, with higher scores indicating greater adherence. Detailed information on the calculation of the PDI is provided in Supplementary Table S2.

### 2.6 Statistical analysis

Descriptive statistics were used to summarize the baseline characteristics of the study population. Continuous variables were expressed as mean ± standard deviation (SD), and categorical variables were presented as frequencies and percentages. Group differences were assessed using the Chi-square test or the Mann–Whitney U test. Spearman’s rank correlation coefficients (*r*_s_) were used to assess correlations among air pollutant concentrations. To examine the associations between long-term air pollution exposure and the risks of IBD, CD, and UC, we applied a time-dependent Cox regression model adjusted for age, sex, ethnicity, BMI, employment status, educational qualifications, household income, physical activity, smoking status, and alcohol consumption. Missing values in categorical covariates were handled by assigning participants to a separate “unknown” category for each respective variable (36, 44). Time-weighted air pollution concentrations and age were incorporated into the Cox models as time-varying covariates with a 1-year time scale to account for temporal changes in exposure levels and aging during follow-up. The model structure followed established methodological approaches described in previous studies (36, 45). Hazard ratios (HRs) and 95% confidence intervals (CIs) associated with each interquartile range (IQR) increase in pollutant concentrations were estimated to quantify the exposure–response relationship. To further explore potential nonlinear associations between air pollution exposure and disease outcomes, restricted cubic spline (RCS) functions were employed to flexibly characterize the exposure-response relationship between long-term air pollutant exposure and IBD risk. Knot points were placed at the 10th, 50th, and 90th percentiles of the exposure distribution. To assess the potential modifying effect of PDI on the associations between air pollution exposure and the risk of IBD and its subtypes, stratified analyses were performed according to PDI scores. We further evaluated multiplicative interaction by including a product term between air pollution exposure and PDI in the time-dependent Cox regression model, with significance assessed using the likelihood ratio test.

Sensitivity analyses were conducted to assess the robustness of the results: (1) excluding participants diagnosed with IBD within the first 2 years of follow-up to minimize the potential influence of reverse causation; (2) excluding participants diagnosed with IBD within the first 5 years of follow-up to evaluate the influence of a longer latency period; (3) additionally adjusting for the use of proton pump inhibitors (PPIs) or non-steroidal anti-inflammatory drugs (NSAIDs), as well as vitamin, mineral, or dietary supplement use, to assess whether the observed associations were influenced by medication and supplement-related confounding; (4) performing multiple imputation by chained equations for covariates with missing values to assess potential bias arising from missing data; and (5) incorporating different air pollutants to establish two-pollutant models.

All analyses were performed using R software (version 4.2.2). Time-dependent Cox regression models were implemented using the “survival” package, with the tmerge() function used to construct time-dependent covariates and the coxph() function used to fit Cox models. RCS modeling was conducted using the “rms” package, and multiple imputation by chained equations was performed using the “mice” package. All statistical tests were two-sided, and a *p* < 0.05 was considered statistically significant.

## 3 Result

### 3.1 Baseline characteristics

Table 1 presents the baseline characteristics of the participants. During a median follow-up of 13.23 years, a total of 1,037 incident IBD cases were identified, including 370 CD and 731 UC cases, among which 64 participants had coexisting CD and UC. The mean age of all participants was 55.67 years, and 55.9% were female. Participants who developed IBD during follow-up had slightly higher overall BMI and a greater proportion of overweight or obese individuals compared with those who did not develop IBD. In terms of socioeconomic characteristics, participants who developed IBD generally had lower educational attainment and household income, a slightly lower proportion engaged in paid employment or self-employment, and a slightly higher proportion of retirees. Regarding lifestyle factors, participants with IBD had a higher proportion of former or current smokers, the majority remained current drinkers, and the proportion with low physical activity levels was also relatively higher. Moreover, participants with IBD exhibited higher exposure levels to most air pollutants and a relatively greater proportion had low adherence to a plant-based diet.

**Table 1:** Baseline characteristics of the included participants.

Characteristics	Overall	IBD			CD			UC		
		No	Yes	*p*-value	No	Yes	*p*-value	No	Yes	*p*-value
*N*	165,722	164,685	1,037		165,352	370		164,991	731	
Age, mean (SD), years	55.67 (7.95)	55.67 (7.95)	55.82 (7.93)	0.472	55.67 (7.95)	55.55 (7.89)	0.779	55.67 (7.95)	55.85 (7.99)	0.467
Sex, *n* (%)										
Male	73,048 (44.1%)	72,531 (44.0%)	517 (49.9%)	<0.001	72,873 (44.1%)	175 (47.3%)	0.212	72,679 (44.1%)	369 (50.5%)	<0.001
Female	92,674 (55.9%)	92,154 (56.0%)	520 (50.1%)		92,479 (55.9%)	195 (52.7%)		92,312 (55.9%)	362 (49.5%)	
Ethnicity, *n* (%)										
White	150,468 (90.8%)	149,516 (90.8%)	952 (91.8%)	0.198	150,133 (90.8%)	335 (90.5%)	0.976	149,791 (90.8%)	677 (92.6%)	0.073
Other	15,190 (9.2%)	15,107 (9.2%)	83 (8.0%)		15,156 (9.2%)	34 (9.2%)		15,137 (9.2%)	53 (7.3%)	
Unknown	64 (0.0%)	62 (0.0%)	2 (0.2%)		63 (0.0%)	1 (0.3%)		63 (0.0%)	1 (0.1%)	
Years of education, *n* (%)										
≤7	12,447 (7.5%)	12,345 (7.5%)	102 (9.8%)	0.004	12,414 (7.5%)	33 (8.9%)	0.687	12,374 (7.5%)	73 (10.0%)	0.004
8–10	40,805 (24.6%)	40,552 (24.6%)	253 (24.4%)		40,716 (24.6%)	89 (24.1%)		40,622 (24.6%)	183 (25.0%)	
11–13	22,209 (13.4%)	22,055 (13.4%)	154 (14.9%)		22,153 (13.4%)	56 (15.1%)		22,100 (13.4%)	109 (14.9%)	
14–15	7,944 (4.8%)	7,884 (4.8%)	60 (5.8%)		7,925 (4.8%)	19 (5.1%)		7,898 (4.8%)	46 (6.3%)	
16–19	8,471 (5.1%)	8,415 (5.1%)	56 (5.4%)		8,451 (5.1%)	20 (5.4%)		8,433 (5.1%)	38 (5.2%)	
≥20	73,083 (44.1%)	72,678 (44.1%)	405 (39.1%)		72,933 (44.1%)	150 (40.5%)		72,806 (44.1%)	277 (37.9%)	
Unknown	763 (0.5%)	756 (0.5%)	7 (0.7%)		760 (0.5%)	3 (0.8%)		758 (0.5%)	5 (0.7%)	
Household income, *n* (%)										
≤£30,999	55,836 (33.7%)	55,467 (33.7%)	369 (35.6%)	0.077	55,713 (33.7%)	123 (33.2%)	0.908	55,568 (33.7%)	268 (36.7%)	0.023
>£30,999	93,196 (56.2%)	92,649 (56.3%)	547 (52.7%)		92,988 (56.2%)	208 (56.2%)		92,823 (56.3%)	373 (51.0%)	
Unknown	16,690 (10.1%)	16,569 (10.1%)	121 (11.7%)		16,651 (10.1%)	39 (10.5%)		16,600 (10.1%)	90 (12.3%)	
Employment status, *n* (%)										
In paid employment or self-employed	104,316 (62.9%)	103,668 (62.9%)	648 (62.5%)	0.815	104,083 (62.9%)	233 (63.0%)	0.983	103,858 (62.9%)	458 (62.7%)	0.986
Retired	49,331 (29.8%)	49,015 (29.8%)	316 (30.5%)		49,221 (29.8%)	110 (29.7%)		49,112 (29.8%)	219 (30.0%)	
Unemployed	11,638 (7.0%)	11,569 (7.0%)	69 (6.7%)		11,613 (7.0%)	25 (6.8%)		11,586 (7.0%)	52 (7.1%)	
Unknown	437 (0.3%)	433 (0.3%)	4 (0.4%)		435 (0.3%)	2 (0.5%)		435 (0.3%)	2 (0.3%)	
Smoking status, *n* (%)										
Never	95,488 (57.6%)	94,987 (57.7%)	501 (48.3%)	<0.001	95,300 (57.6%)	188 (50.8%)	0.024	95,149 (57.7%)	339 (46.4%)	<0.001
Previous	57,185 (34.5%)	56,754 (34.5%)	431 (41.6%)		57,040 (34.5%)	145 (39.2%)		56,867 (34.5%)	318 (43.5%)	
Current	12,637 (7.6%)	12,535 (7.6%)	102 (9.8%)		12,601 (7.6%)	36 (9.7%)		12,565 (7.6%)	72 (9.8%)	
Unknown	412 (0.2%)	409 (0.2%)	3 (0.3%)		411 (0.2%)	1 (0.3%)		410 (0.2%)	2 (0.3%)	
Drinking status, *n* (%)										
Never	5,015 (3.0%)	4,980 (3.0%)	35 (3.4%)	0.190	4,999 (3.0%)	16 (4.3%)	0.041	4,990 (3.0%)	25 (3.4%)	0.238
Previous	4,390 (2.6%)	4,354 (2.6%)	36 (3.5%)		4,374 (2.6%)	16 (4.3%)		4,364 (2.6%)	26 (3.6%)	
Current	156,172 (94.2%)	155,210 (94.2%)	962 (92.8%)		155,835 (94.2%)	337 (91.1%)		155,495 (94.2%)	677 (92.6%)	
Unknown	145 (0.1%)	141 (0.1%)	4 (0.4%)		144 (0.1%)	1 (0.3%)		142 (0.1%)	3 (0.4%)	
Physical activity, *n* (%)										
Low	24,456 (14.8%)	24,280 (14.7%)	176 (17.0%)	0.100	24,394 (14.8%)	62 (16.8%)	0.274	24,334 (14.7%)	122 (16.7%)	0.256
Moderate	58,544 (35.3%)	58,178 (35.3%)	366 (35.3%)		58,401 (35.3%)	143 (38.6%)		58,300 (35.3%)	244 (33.4%)	
High	54,624 (33.0%)	54,302 (33.0%)	322 (31.1%)		54,512 (33.0%)	112 (30.3%)		54,388 (33.0%)	236 (32.3%)	
Unknown	28,098 (17.0%)	27,925 (17.0%)	173 (16.7%)		28,045 (17.0%)	53 (14.3%)		27,969 (17.0%)	129 (17.6%)	
BMI, (kg/m^2^), *n* (%)										
<18.5	904 (0.5%)	896 (0.5%)	8 (0.8%)	0.138	899 (0.5%)	5 (1.4%)	0.098	900 (0.5%)	4 (0.5%)	0.085
18.5–24.9	63,288 (38.2%)	62,926 (38.2%)	362 (34.9%)		63,154 (38.2%)	134 (36.2%)		63,042 (38.2%)	246 (33.7%)	
25.0–29.9	68,303 (41.2%)	67,855 (41.2%)	448 (43.2%)		68,156 (41.2%)	147 (39.7%)		67,975 (41.2%)	328 (44.9%)	
≥30.0	33,227 (20.0%)	33,008 (20.0%)	219 (21.1%)		33,143 (20.0%)	84 (22.7%)		33,074 (20.0%)	153 (20.9%)	
Air pollutant exposure, mean (SD), μg/m^3^										
PM_2.5_	10.87 (2.2)	10.87 (2.2)	10.97 (2.12)	0.190	10.87 (2.2)	10.89 (2.09)	0.774	10.87 (2.2)	11.02 (2.15)	0.086
PM_10_	16.08 (2.67)	16.08 (2.67)	16.24 (2.57)	0.048	16.08 (2.67)	16.16 (2.5)	0.294	16.08 (2.67)	16.29 (2.61)	0.051
NO_x_	32.05 (13.8)	32.04 (13.8)	32.48 (13.28)	0.087	32.05 (13.8)	32.15 (13.5)	0.680	32.04 (13.8)	32.74 (13.14)	0.035
NO_2_	20.64 (7.22)	20.64 (7.22)	20.92 (6.96)	0.102	20.64 (7.22)	20.71 (7.04)	0.699	20.64 (7.22)	21.07 (6.92)	0.041
SO_2_	2.63 (1.48)	2.63 (1.48)	2.69 (1.57)	0.099	2.63 (1.48)	2.7 (1.52)	0.246	2.63 (1.48)	2.67 (1.57)	0.345
Benzene	0.52 (0.15)	0.52 (0.15)	0.52 (0.14)	0.091	0.52 (0.15)	0.52 (0.14)	0.622	0.52 (0.15)	0.53 (0.15)	0.034
Adherence to PDI, *n* (%)										
Low (≤49)	57,283 (34.6%)	56,909 (34.6%)	374 (36.1%)	0.302	57,170 (34.6%)	113 (30.5%)	0.263	57,007 (34.6%)	276 (37.8%)	0.112
Moderate (50–54)	60,355 (36.4%)	60,001 (36.4%)	354 (34.1%)		60,213 (36.4%)	142 (38.4%)		60,113 (36.4%)	242 (33.1%)	
High (≥55)	48,084 (29.0%)	47,775 (29.0%)	309 (29.8%)		47,969 (29.0%)	115 (31.1%)		47,871 (29.0%)	213 (29.1%)	

IBD, inflammatory bowel disease; UC, ulcerative colitis; CD, Crohn’s disease; BMI, body mass index; PDI, Plant-based Diet Index; SD, standard deviation; PM2.5, particulate matter with aerodynamic diameter <2.5 μm; PM10, particulate matter with aerodynamic diameter <10 μm; NO_2_, nitrogen dioxide; NOx, nitrogen oxides; SO2, sulfur dioxide. Data are presented as mean (SD) for continuous variables and number (percentage) for categorical variables. *p*-values were reported for descriptive and exploratory purposes only.

Spearman’s rank correlation analysis revealed a broad pattern of positive correlations among ambient air pollutants, with the strongest correlations observed between PM_2.5_ and PM_10_ (*r*_s_ = 0.943) and between NO_x_ and NO_2_ (*r*_s_ = 0.999). Other pollutants also showed varying degrees of positive correlations (*r*_s_ = 0.215–0.833), whereas SO_2_ exhibited relatively weak correlations with the other pollutants (Supplementary Figure S1).

### 3.2 Association between air pollution and risk of IBD

**Figure 2:**
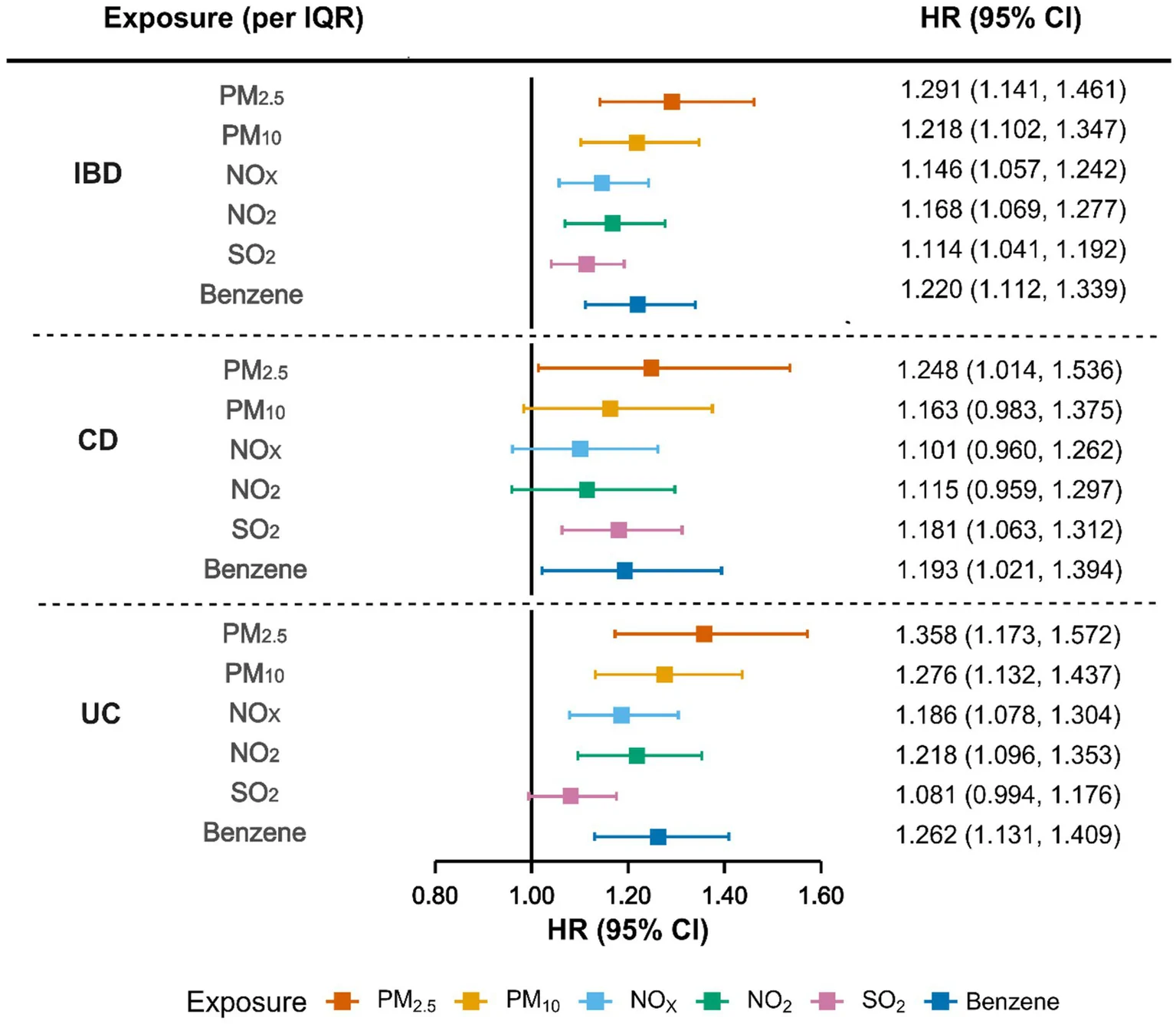
Hazard ratios (HRs) and 95% confidence intervals (CIs) for the incidence of IBD (inflammatory bowel disease), CD (Crohn’s disease), and UC (ulcerative colitis) associated with per interquartile range (IQR) increase in air pollutants (PM_2.5_, particulate matter with aerodynamic diameter <2.5 μm; PM10, particulate matter with aerodynamic diameter <10 μm; NO_2_, nitrogen dioxide; NOx, nitrogen oxides; SO2, sulfur dioxide; benzene). Time-varying Cox regression model was used to evaluate the associations between each exposure and disease incidence, adjusting for potential confounders including age, sex, ethnicity, BMI, employment status, educational qualifications, household income, physical activity, smoking status, and alcohol consumption status.

Based on the time-dependent Cox regression model with adjustment for potential confounders, increased exposure levels to PM_2.5_, PM_10_, NO_x_, NO_2_, SO_2_, and benzene were all significantly associated with an elevated risk of IBD. For each IQR increase in pollutant concentration, the corresponding HRs and 95% CIs were as follows: PM_2.5_, 1.291 (1.141–1.461); PM_10_, 1.218 (1.102–1.347); NO_x_, 1.146 (1.057–1.242); NO_2_, 1.168 (1.069–1.277); SO_2_, 1.114 (1.041–1.192); and benzene, 1.220 (1.112–1.339). Across both IBD subtypes (CD and UC), air pollutant exposures were positively associated with disease risk. For CD, only PM_2.5_, SO_2_ and benzene showed significant associations, with HRs of 1.248 (1.014–1.536), 1.181 (1.063–1.312), and 1.193 (1.021–1.394), respectively. For UC, significant associations were observed for PM_2.5_, PM_10_, NO_x_, NO_2_, and benzene, with HRs of 1.358 (1.173–1.572), 1.276 (1.132–1.437), 1.186 (1.078–1.304), 1.218 (1.096–1.353), and 1.262 (1.131–1.409), respectively (Figure 2). In addition, results of the sensitivity analyses were generally consistent with the main findings, further supporting the robustness of the observed associations (Supplementary Tables S3–S5).

### 3.3 Exposure–response relationships between ambient pollutants and IBD risk

**Figure 3:**
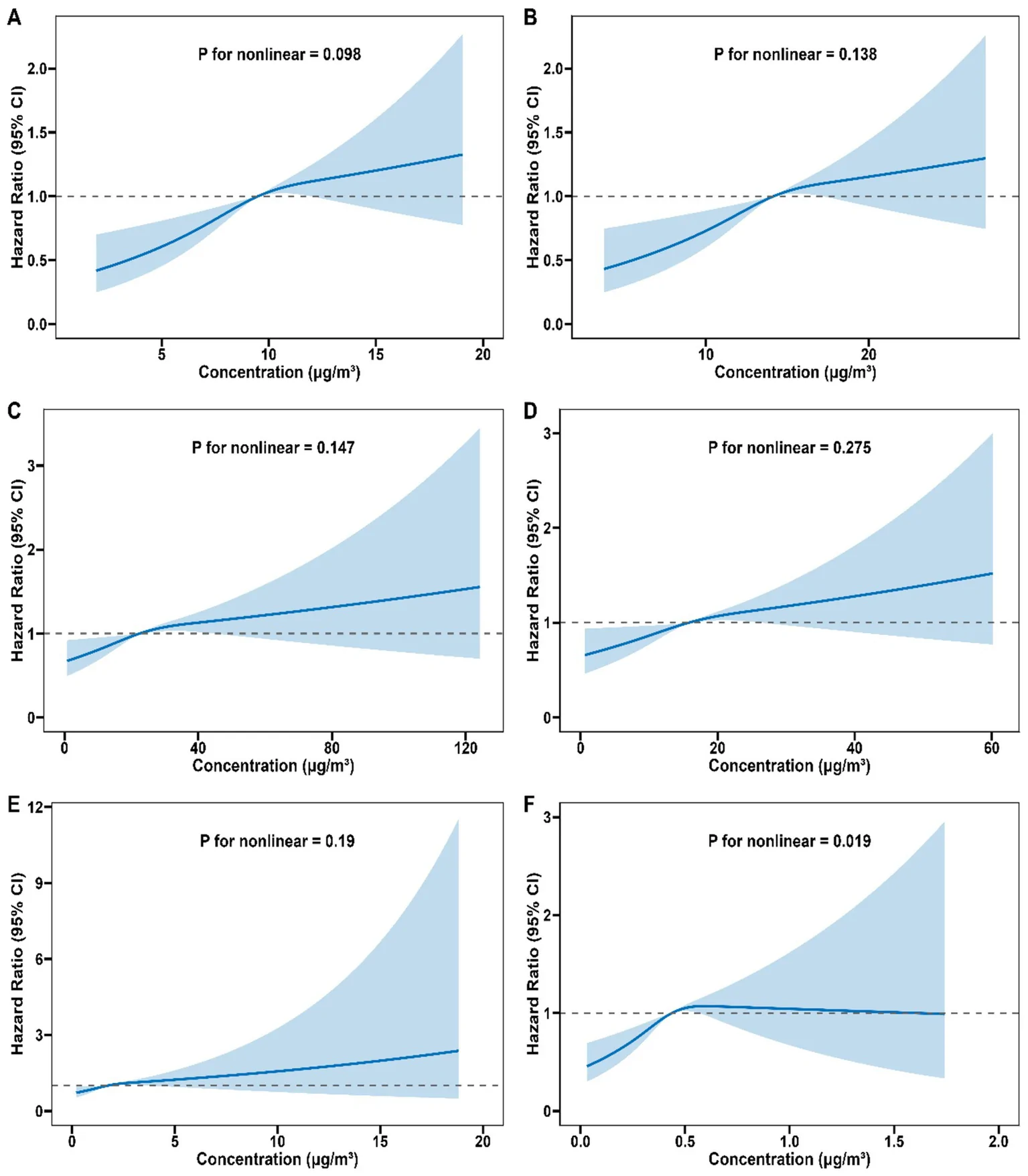
Exposure–response relationships between ambient air pollutants and the risk of inflammatory bowel disease. Models were adjusted for age, sex, ethnicity, BMI, employment status, education, household income, physical activity, smoking status, and alcohol consumption. Panels A–F correspond to the following pollutants: **(A)** PM_2.5_, particulate matter with aerodynamic diameter <2.5 μm, **(B)** PM_10_, particulate matter with aerodynamic diameter <10 μm, **(C)** NO_x_, nitrogen oxides, **(D)** NO_2_, nitrogen dioxide, **(E)** SO_2_, sulfur dioxide, and **(F)** benzene.

As shown in Figure 3, air pollution exposure exhibited an approximately linear and monotonically increasing relationship with IBD risk (all non-linearity *p*-values > 0.05), except for the benzene (non-linearity *p*-value = 0.019). In the subtype analysis, CD showed a consistent pattern with the IBD. For UC, exposures to PM_2.5_, PM_10_, NO_x_, NO_2_, and benzene were associated with linear increases in disease risk, but not for SO_2_ (non-linearity *p*-value = 0.04; Supplementary Figures S2, S3).

### 3.4 Effect modification by PDI on the air pollution–IBD association

**Figure 4:**
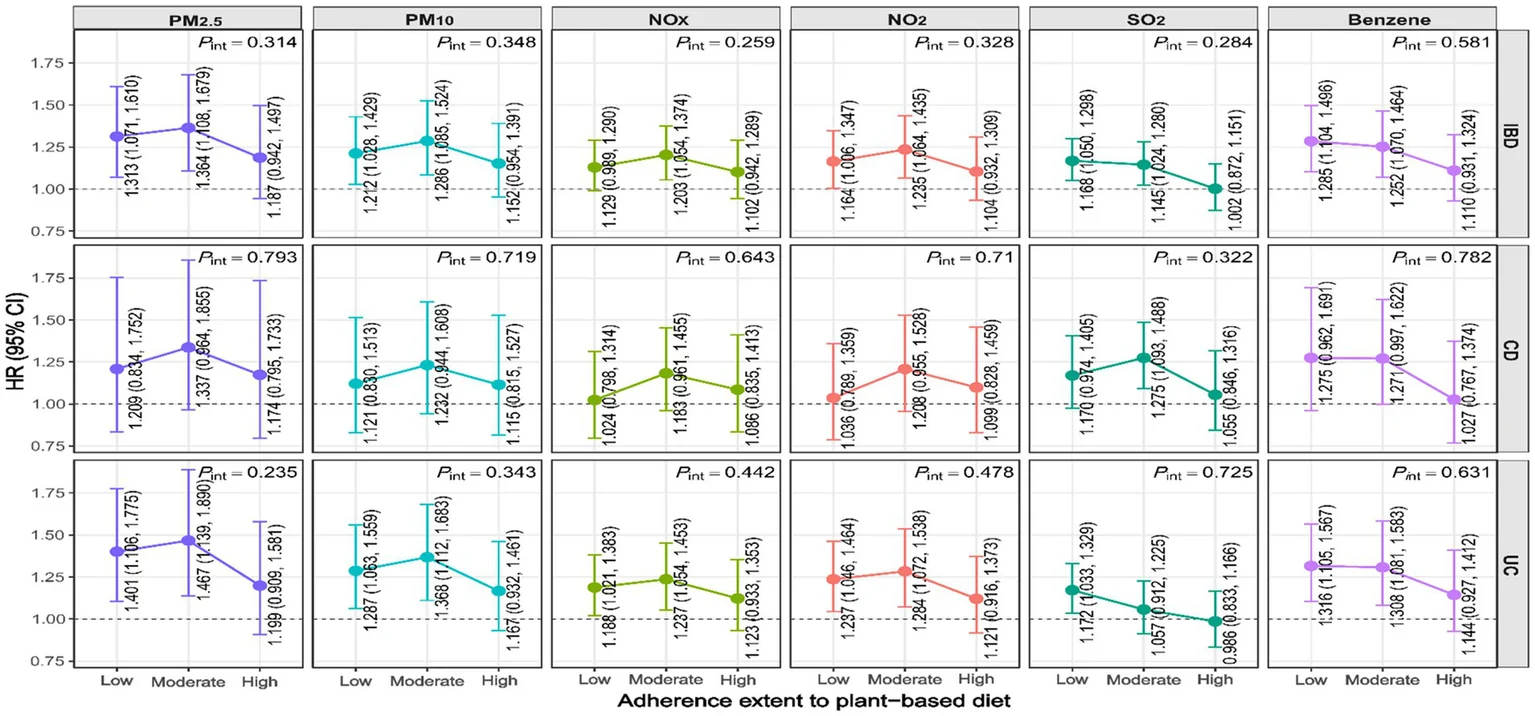
Associations between air pollutants (PM_2.5_, particulate matter with aerodynamic diameter <2.5 μm; PM10, particulate matter with aerodynamic diameter <10 μm; NO2, nitrogen dioxide; NOx, nitrogen oxides; SO2, sulfur dioxide) and the risk of IBD (inflammatory bowel disease), CD (Crohn’s disease), and UC (ulcerative colitis) across different levels of adherence to a plant-based diet. HRs and 95% CIs were estimated using time-dependent Cox regression model. Models were adjusted for age, sex, ethnicity, BMI, employment status, education, household income, physical activity, smoking status, and alcohol consumption.

The stratified analysis showed that the associations between air pollution and IBD varied across different PDI levels (Figure 4). Among participants with low PDI, air pollution was significantly associated with an increased risk of IBD, with the corresponding HRs (95% CIs) as follows: PM_2.5_, 1.313 (1.071–1.61); PM_10_, 1.212 (1.028–1.429); NO_2_, 1.164 (1.006–1.347); SO_2_, 1.168 (1.05–1.298); and benzene, 1.285 (1.104–1.496). In contrast, among participants with high PDI, the point estimates for the associations between air pollutants and IBD risk were smaller and no longer statistically significant. A similar pattern was observed in the subtype analysis of IBD. For instance, air pollutants were significantly associated with an increased risk of UC among participants in the low PDI group, with corresponding HRs (95% CIs) of: PM_2.5_, 1.401 (1.106–1.775); PM_10_, 1.287 (1.063–1.559); NO_x_, 1.188(1.021–1.383); NO_2_, 1.237 (1.046–1.464); SO_2_, 1.172 (1.033–1.329); and benzene, 1.316 (1.105–1.567). In the high PDI group, however, the point estimates for the associations between air pollutants and UC risk were smaller and no longer statistically significant. It should be noted, however, that no statistically significant interaction was observed between air pollution exposure and PDI in relation to the risk of IBD or its subtypes (all *p* for interaction > 0.05).

## 4 Discussion

Based on the large-scale prospective UK Biobank cohort, this study systematically evaluated the association between long-term exposure to ambient air pollution and the risk of developing IBD, and further examined the potential modifying role of plant-based diet in this relationship. Multiple air pollutants (PM_2.5_, PM_10_, NO_x_, NO_2_, SO_2_, and benzene) were found to be significantly associated with an increased risk of IBD. In subtype analyses, the risk of CD was primarily associated with exposure to PM_2.5,_ SO_2_ and benzene, whereas the risk of UC was significantly associated with exposure to PM_2.5_, PM_10_, NO_x_, NO_2_ and benzene. Notably, compared with individuals with lower PDI, the associations between air pollution exposure and IBD risk appeared weaker and were no longer statistically significant among those with higher PDI. A similar pattern was also observed for UC.

Existing evidence has suggested a potential link between air pollution and IBD; however, epidemiological studies on this association remain limited. For example, a study based on the UK Biobank reported that environmental benzene exposure was associated with an increased risk of IBD (18), and a large cohort study from Italy similarly found that PM_2.5_ exposure significantly increased the incidence of IBD (14). Building upon these earlier findings, our study incorporated a broader range of pollutant indicators and systematically evaluated the effects of multiple air pollutants. We found that long-term exposures to PM_2.5_, PM_10_, NO_x_, NO_2_, SO_2_, and benzene were all associated with an increased risk of IBD, further supporting the notion that air pollution may be an important environmental risk factor for IBD. Previous studies have shown that air pollution exposure can alter gut microbial composition, induce oxidative stress, disrupt lipid metabolism, and increase intestinal permeability, thereby triggering chronic immune–inflammatory responses. These biological processes may collectively contribute to the development and progression of IBD (46).

Concerning the IBD subtypes, evidence regarding exposure to air pollutants has primarily focused on particulate matter and nitrogen oxides, while research on benzene and sulfur dioxide remains relatively limited. To date, only one study based on the UK Biobank has confirmed that benzene exposure was associated with increased risks of both UC and CD (18). To the best of our knowledge, the present study is the first large-scale prospective cohort investigation to systematically assess the impact of long-term SO_2_ exposure on IBD subtypes. Our findings indicate that SO_2_ exposure was significantly associated with an increased risk of CD. However, the findings across studies are not entirely consistent. These inconsistencies may stem from differences in geographic regions, levels of environmental pollution, sample sizes, and exposure assessment methods. For example, a previous study reported that exposure to PM_2.5_, PM_10_, and NO_2_ was associated with an elevated risk of UC but not CD (47); a case–control study reported that NO_2_ exposure may increase CD risk in younger populations (48); and the EPIC cohort in Europe observed no significant association between air pollution and either CD or UC (17). Building upon the existing evidence, our study provides several important advancements. Leveraging the large prospective UK Biobank cohort and long-term follow-up, and applying time-dependent Cox models that incorporate dynamically updated residential exposure levels, we were able to more accurately characterize individual long-term air pollution exposure and reduce exposure misclassification bias. Notably, our study observed a relatively greater increase in UC risk associated with air pollution exposure, whereas the corresponding increase for CD appeared less pronounced, suggesting that UC may be more susceptible to air pollution. Twin studies have shown that the concordance rate for CD in monozygotic twins can reach 55%, markedly higher than the approximately 17% observed for UC, while concordance rates in dizygotic twins remain low for both diseases (49–52). These findings suggest a stronger genetic contribution to CD development.

Furthermore, our findings suggested that the association between air pollution exposure and the risk of IBD may be weaker among participants with higher adherence to a plant-based diet. The pattern of relatively lower risk estimates across higher PDI strata remained noteworthy. This pattern suggests that a plant-based diet may be associated with lower susceptibility to the adverse effects of air pollution on IBD risk. Several mechanisms may help explain these observations. Plant-based diet is typically rich in dietary fiber, which can enhance gut microbial diversity, promote mucosal barrier repair, and reduce the risk of developing IBD (53–55). In addition, polyphenols, carotenoids, and other phytochemicals abundant in plant-derived foods possess strong antioxidant and anti-inflammatory properties, helping to suppress oxidative stress, reduce pro-inflammatory cytokine release, and improve intestinal barrier integrity (56). Given that oxidative stress and mucosal inflammation are key pathways through which air pollution affects gut health, a higher PDI may help explain the observed pattern of associations between air pollution exposure and IBD risk.

In the stratified analyses across different PDI levels, we found that the association between air pollution and IBD risk appeared to be more evident in UC. With increasing PDI, the risk of UC showed a more pronounced decreasing trend. This difference may be related to the distinct lesion locations and underlying pathophysiological mechanisms of UC and CD. CD can involve any segment of the gastrointestinal tract, whereas UC primarily affects the colonic mucosa (3). Colon contains approximately 38 trillion bacteria—far more than any other part of the human body—and represents the most densely colonized region of the gut microbiota (57). Plant-based diets are rich in dietary fiber, which can be metabolized by colonic microbiota to produce short-chain fatty acids and other metabolites with anti-inflammatory properties and functions that help maintain the integrity of the intestinal mucosal barrier (58). Therefore, in this highly microbiota-enriched local environment, the beneficial metabolic processes associated with a plant-based diet may help alleviate inflammatory responses triggered by air pollution exposure, which may partly explain the relatively weaker observed association between air pollution exposure and UC risk.

Although our findings suggest that greater adherence to a plant-based dietary pattern may provide additional benefits, the interaction tests did not reach statistical significance; therefore, the observed dietary modification effects should be interpreted cautiously as suggestive findings. From a public health perspective, our findings highlight the importance of population-level strategies to reduce ambient air pollution exposure in lowering the environmental burden of IBD. Given that air pollution exposure is widespread and largely involuntary, regulatory measures aimed at improving air quality, such as reducing industrial and traffic-related emissions and strengthening ambient air quality standards, remain essential for population-level prevention. Within this framework, dietary patterns may represent one component of broader preventive strategies to help buffer inflammation-related damage associated with environmental exposures. A modelling study further suggests that integrated strategies combining agricultural emission reductions with dietary transitions may yield greater population health benefits than either approach alone (59). Therefore, integrating dietary guidance with environmental governance may provide a broader public health framework for IBD prevention, while effective reduction of air pollution exposure should remain the primary policy priority.

This study still has several limitations. First, it is based on participants from the UK Biobank, the majority of whom are White; therefore, the generalizability of the findings to other ethnic groups and populations with different sociocultural backgrounds may be limited. Second, dietary information was collected using repeated 24-h dietary recalls, which are considered relatively reliable and less prone to recall bias. Although averaging up to five recalls collected over multiple years may improve the estimation of habitual dietary intake, this approach may still not fully capture long-term stable dietary patterns. Moreover, dietary intake was treated as fixed during follow-up, so potential changes in diet over time could not be accounted for, potentially leading to exposure misclassification. In addition, the UK Biobank is subject to a “healthy volunteer” selection bias, as participants are generally healthier and of higher socioeconomic status than the general population (60). This may limit the generalizability of our findings and influence the estimated HRs. If the relatively healthy cohort had narrower exposure contrasts and lower baseline IBD risk, the observed HRs may have been biased toward the null, potentially underestimating the true associations, although the direction and magnitude of this bias remain uncertain. Finally, as an observational study, although we adjusted for multiple covariates and conducted sensitivity analyses to enhance the robustness of our findings, the possibility of residual confounding cannot be entirely ruled out. Therefore, further independent validation in diverse populations, along with longitudinal intervention and experimental studies, is warranted to elucidate the causal effects of plant-based diet on air pollution–related IBD risk and to uncover the underlying biological mechanisms.

## 5 Conclusion

In summary, long-term exposure to ambient air pollution is associated with an increased risk of IBD, and higher adherence to a plant-based diet was associated with a relatively lower air pollution-related IBD risk, with particularly pronounced associations observed for UC. These findings underscore the importance of jointly considering both environmental and lifestyle factors in public health approaches to understanding and managing IBD risk. Future studies integrating microbiome and metabolomic data are warranted to further validate and elucidate the biological pathways linking pollution exposure, dietary patterns, and intestinal inflammation.

## Data Availability

The data analyzed in this study is subject to the following licenses/restrictions: the data used in this study are not publicly available due to access restrictions. Data are available from the UK Biobank upon reasonable request and approval of a formal application. Researchers can apply for access through the UK Biobank Access Management System. Requests to access these datasets should be directed to https://www.ukbiobank.ac.uk/.

## Footnotes

1
https://biobank.ctsu.ox.ac.uk/crystal/crystal/docs/DietWebQ.pdf

## References

[1] Buie MJ Quan J Windsor JW Coward S Hansen TM King JA . Global hospitalization trends for Crohn's disease and ulcerative colitis in the 21st century: a systematic review with temporal analyses. Clin Gastroenterol Hepatol. (2023) 21:2211–21. doi: 10.1016/j.cgh.2022.06.030, 35863682

[2] Kaplan GG. The global burden of IBD: from 2015 to 2025. Nat Rev Gastroenterol Hepatol. (2015) 12:720–7. doi: 10.1038/nrgastro.2015.150, 26323879

[3] Kobayashi T Siegmund B Le Berre C Wei SC Ferrante M Shen B . Ulcerative colitis. Nat Rev Dis Primers. (2020) 6:74. doi: 10.1038/s41572-020-0205-x 32913180

[4] Khor B Gardet A Xavier RJ. Genetics and pathogenesis of inflammatory bowel disease. Nature. (2011) 474:307–17. doi: 10.1038/nature10209, 21677747 PMC3204665

[5] Ordás I Eckmann L Talamini M Baumgart DC Sandborn WJ. Ulcerative colitis. Lancet. (2012) 380:1606–19. doi: 10.1016/s0140-6736(12)60150-0, 22914296

[6] Piovani D Danese S Peyrin-Biroulet L Nikolopoulos GK Lytras T Bonovas S. Environmental risk factors for inflammatory bowel diseases: an umbrella review of Meta-analyses. Gastroenterology. (2019) 157:647–59.e4. doi: 10.1053/j.gastro.2019.04.016, 31014995

[7] Landrigan PJ Fuller R Acosta NJR Adeyi O Arnold R Basu NN . The lancet commission on pollution and health. Lancet. (2018) 391:462–512. doi: 10.1016/s0140-673(17)32345-0 29056410

[8] Institute HE. State of Global Air 2025: A Report on Air Pollution and its Role in the World’s Leading Causes of Death. Boston, MA: Health Effects Institute (2025).

[9] Turner MC Andersen ZJ Baccarelli A Diver WR Gapstur SM Pope CA 3rd . Outdoor air pollution and cancer: an overview of the current evidence and public health recommendations. CA Cancer J Clin. (2020) 70:460–79. doi: 10.3322/caac.21632, 32964460 PMC7904962

[10] Li Y Xu L Shan Z Teng W Han C. Association between air pollution and type 2 diabetes: an updated review of the literature. Ther Adv Endocrinol Metab. (2019) 10:2042018819897046. doi: 10.1177/2042018819897046, 31903180 PMC6931138

[11] Laumbach RJ Kipen HM. Respiratory health effects of air pollution: update on biomass smoke and traffic pollution. J Allergy Clin Immunol. (2012) 129:3–11. doi: 10.1016/j.jaci.2011.11.021, 22196520 PMC3272333

[12] Al-Kindi SG Brook RD Biswal S Rajagopalan S. Environmental determinants of cardiovascular disease: lessons learned from air pollution. Nat Rev Cardiol. (2020) 17:656–72. doi: 10.1038/s41569-020-0371-2, 32382149 PMC7492399

[13] Kou Y Du S Du W Ye W Yang Y Qin L. Exposure to air pollution and non-neoplastic digestive system diseases: findings from the China health and retirement longitudinal study. Front Public Health. (2024) 12:1372156. doi: 10.3389/fpubh.2024.1372156, 39618951 PMC11604608

[14] Fu C Wang Q Chen Y Zhang Y. Exploring the causal relationship between airborne particulate matter and ulcerative colitis: a two-sample Mendelian randomization study. PLoS One. (2024) 19:e0300066. doi: 10.1371/journal.pone.0300066, 38457365 PMC10923436

[15] Adami G Pontalti M Cattani G Rossini M Viapiana O Orsolini G . Association between long-term exposure to air pollution and immune-mediated diseases: a population-based cohort study. RMD Open. (2022) 8:e002055. doi: 10.1136/rmdopen-2021-002055, 35292563 PMC8969049

[16] Chen J Dan L Sun Y Yuan S Liu W Chen X . Ambient air pollution and risk of enterotomy, gastrointestinal cancer, and all-cause mortality among 4,708 individuals with inflammatory bowel disease: a prospective cohort study. Environ Health Perspect. (2023) 131:77010. doi: 10.1289/ehp12215, 37505744 PMC10379095

[17] Opstelten JL Beelen RMJ Leenders M Hoek G Brunekreef B van Schaik FDM . Exposure to ambient air pollution and the risk of inflammatory bowel disease: a European nested case-control study. Dig Dis Sci. (2016) 61:2963–71. doi: 10.1007/s10620-016-4249-4, 27461060 PMC5020109

[18] Xiao ZH Huang S Zhao K Zhang X Li Z Li R . Association between long-term benzene exposure and inflammatory bowel disease in a national cohort: the modifying effect of genetic susceptibility. Ecotoxicol Environ Saf. (2025) 296:118198. doi: 10.1016/j.ecoenv.2025.118198, 40239550

[19] Schwingshackl L Bogensberger B Hoffmann G. Diet quality as assessed by the healthy eating index, alternate healthy eating index, dietary approaches to stop hypertension score, and health outcomes: an updated systematic review and meta-analysis of cohort studies. J Acad Nutr Diet. (2018) 118:74–100.e11. doi: 10.1016/j.jand.2017.08.024, 29111090

[20] Bahari H Taheri S Rashidmayvan M Jamshidi S Jazinaki MS Pahlavani N. The effect of royal jelly on liver enzymes and glycemic indices: a systematic review and meta-analysis of randomized clinical trials. Complement Ther Med. (2023) 77:102974. doi: 10.1016/j.ctim.2023.102974, 37619715

[21] Jandari S Ghavami A Ziaei R Nattagh-Eshtivani E Rezaei Kelishadi M Sharifi S . Effects of *Momordica charantia* L on blood pressure: a systematic review and meta- analysis of randomized clinical trials. Int J Food Prop. (2020) 23:1913–24. doi: 10.1080/10942912.2020.1833916

[22] Wang Y Liu B Han H Hu Y Zhu L Rimm EB . Associations between plant-based dietary patterns and risks of type 2 diabetes, cardiovascular disease, cancer, and mortality - a systematic review and meta-analysis. Nutr J. (2023) 22:46. doi: 10.1186/s12937-023-00877-2, 37789346 PMC10548756

[23] Chew HSJ Heng FKX Tien SA Thian JY Chou HS Loong SSE . Effects of plant-based diets on anthropometric and cardiometabolic markers in adults: an umbrella review. Nutrients. (2023) 15:2331. doi: 10.3390/nu15102331, 37242214 PMC10222061

[24] Capodici A Mocciaro G Gori D Landry MJ Masini A Sanmarchi F . Cardiovascular health and cancer risk associated with plant based diets: an umbrella review. PLoS One. (2024) 19:e0300711. doi: 10.1371/journal.pone.0300711, 38748667 PMC11095673

[25] Albenberg LG Lewis JD Wu GD. Food and the gut microbiota in inflammatory bowel diseases: a critical connection. Curr Opin Gastroenterol. (2012) 28:314–20. doi: 10.1097/MOG.0b013e328354586f, 22573192 PMC3822011

[26] Jantchou P Morois S Clavel-Chapelon F Boutron-Ruault MC Carbonnel F. Animal protein intake and risk of inflammatory bowel disease: the E3N prospective study. Am J Gastroenterol. (2010) 105:2195–201. doi: 10.1038/ajg.2010.192, 20461067

[27] Chen H Fu T Dan L Chen X Sun Y Chen J . Meat consumption and all-cause mortality in 5763 patients with inflammatory bowel disease: a retrospective cohort study. EClinicalMedicine. (2022) 47:101406. doi: 10.1016/j.eclinm.2022.101406, 35497068 PMC9046121

[28] Akanyibah FA He C Wang X Wang B Mao F. The role of plant-based dietary compounds in gut microbiota modulation in inflammatory bowel disease. Front Nutr. (2025) 12:1606289. doi: 10.3389/fnut.2025.1606289 40521353 PMC12163340

[29] Chiba M Abe T Tsuda H Sugawara T Tsuda S Tozawa H . Lifestyle-related disease in Crohn's disease: relapse prevention by a semi-vegetarian diet. World J Gastroenterol. (2010) 16:2484–95. doi: 10.3748/wjg.v16.i20.2484 20503448 PMC2877178

[30] Chen J Sun Y Dan L Wellens J Yuan S Yang H . Composition of plant-based diets and the incidence and prognosis of inflammatory bowel disease: a multinational retrospective cohort study. Lancet Reg Health Eur. (2025) 52:101264. doi: 10.1016/j.lanepe.2025.101264, 40166364 PMC11957509

[31] Kelly FJ Fussell JC. Air pollution and public health: emerging hazards and improved understanding of risk. Environ Geochem Health. (2015) 37:631–49. doi: 10.1007/s10653-015-9720-1, 26040976 PMC4516868

[32] Wang B Sun Y Zhang K Wang Y Tan X Wang N . Long-term exposure to ambient air pollution and risk of microvascular complications among patients with type 2 diabetes: a prospective study. Int J Epidemiol. (2024) 53. doi: 10.1093/ije/dyae056, 38632038

[33] Petermann-Rocha F Wirth MD Boonpor J Parra-Soto S Zhou Z Mathers JC . Associations between an inflammatory diet index and severe non-alcoholic fatty liver disease: a prospective study of 171,544 UK biobank participants. BMC Med. (2023) 21:123. doi: 10.1186/s12916-023-02793-y, 37013578 PMC10071692

[34] Sudlow C Gallacher J Allen N Beral V Burton P Danesh J . UK biobank: an open access resource for identifying the causes of a wide range of complex diseases of middle and old age. PLoS Med. (2015) 12:e1001779. doi: 10.1371/journal.pmed.1001779, 25826379 PMC4380465

[35] Collins R. What makes UK biobank special? Lancet. (2012) 379:1173–4. doi: 10.1016/s0140-6736(12)60404-8, 22463865

[36] Yang S Li M Guo C Requia WJ Sakhvidi MJZ Lin K . Associations of long-term exposure to nitrogen oxides with all-cause and cause-specific mortality. Nat Commun. (2025) 16:1730. doi: 10.1038/s41467-025-56963-y, 39966376 PMC11836065

[37] Pugsley KL Stedman JR Brookes DM Kent AJ Morris RJ Whiting SL . Technical Report on UK Supplementary Modelling Assessment under the Air Quality Standards Regulations 2010 for 2020. Manchester, UK: Ricardo Energy & Environment (2022).

[38] Liu B Young H Crowe FL Benson VS Spencer EA Key TJ . Development and evaluation of the Oxford WebQ, a low-cost, web-based method for assessment of previous 24 h dietary intakes in large-scale prospective studies. Public Health `utr. (2011) 14:1998–2005. doi: 10.1017/s1368980011000942, 21729481

[39] Greenwood DC Hardie LJ Frost GS Alwan NA Bradbury KE Carter M . Validation of the Oxford WebQ online 24-hour dietary questionnaire using biomarkers. Am J Epidemiol. (2019) 188:1858–67. doi: 10.1093/aje/kwz165, 31318012 PMC7254925

[40] Zhu K Li R Yao P Yu H Pan A Manson JE . Proteomic signatures of healthy dietary patterns are associated with lower risks of major chronic diseases and mortality. Nat Food. (2025) 6:47–57. doi: 10.1038/s43016-024-01059-x, 39333296

[41] Lv Y Rong S Deng Y Bao W Xia Y Chen L. Plant-based diets, genetic predisposition and risk of non-alcoholic fatty liver disease. BMC Med. (2023) 21:351. doi: 10.1186/s12916-023-03028-w, 37697334 PMC10496397

[42] Zheng X Liu J Wang S Xiao Y Jiang Q Li C . Total physical activity, plant-based diet and neurodegenerative diseases: a prospective cohort study of the UK biobank. Parkinsonism Relat Disord. (2024) 128:107125. doi: 10.1016/j.parkreldis.2024.107125, 39241508

[43] Perez-Cornago A Pollard Z Young H van Uden M Andrews C Piernas C . Description of the updated nutrition calculation of the Oxford WebQ questionnaire and comparison with the previous version among 207,144 participants in UK biobank. Eur J Nutr. (2021) 60:4019–30. doi: 10.1007/s00394-021-02558-4, 33956230 PMC8437868

[44] Papier K Atkins JR Tong TYN Gaitskell K Desai T Ogamba CF . Identifying proteomic risk factors for cancer using prospective and exome analyses of 1463 circulating proteins and risk of 19 cancers in the UK biobank. Nat Commun. (2024) 15:4010. doi: 10.1038/s41467-024-48017-6, 38750076 PMC11096312

[45] Chen Y Yang S Lin J Gu S Wu L Huang W . Long-term exposure to ambient air pollutants and risk of prostate cancer: a prospective cohort study. Environ Res. (2025) 270:121020. doi: 10.1016/j.envres.2025.121020, 39900276

[46] Olstrup H Mohamed HAS Honoré J Schullehner J Sigsgaard T Forsberg B . Air pollution exposure and inflammatory bowel disease: a systematic literature review of epidemiological and mechanistic studies. Front Environ Health. (2024) 3:1463016. doi: 10.3389/fenvh.2024.1463016

[47] Li FR Wu KY Fan WD Chen GC Tian H Wu XB. Long-term exposure to air pollution and risk of incident inflammatory bowel disease among middle and old aged adults. Ecotoxicol Environ Saf. (2022) 242:113835. doi: 10.1016/j.ecoenv.2022.113835, 35816845

[48] Kaplan GG Hubbard J Korzenik J Sands BE Panaccione R Ghosh S . The inflammatory bowel diseases and ambient air pollution: a novel association. Am J Gastroenterol. (2010) 105:2412–9. doi: 10.1038/ajg.2010.252, 20588264 PMC3180712

[49] van Dongen J Slagboom PE Draisma HH Martin NG Boomsma DI. The continuing value of twin studies in the omics era. Nat Rev Genet. (2012) 13:640–53. doi: 10.1038/nrg3243, 22847273

[50] Ng SC Woodrow S Patel N Subhani J Harbord M. Role of genetic and environmental factors in British twins with inflammatory bowel disease. Inflamm Bowel Dis. (2012) 18:725–36. doi: 10.1002/ibd.21747, 21557397

[51] Spehlmann ME Begun AZ Saroglou E Hinrichs F Tiemann U Raedler A . Risk factors in German twins with inflammatory bowel disease: results of a questionnaire-based survey. J Crohns Colitis. (2012) 6:29–42. doi: 10.1016/j.crohns.2011.06.007, 22261525

[52] Halfvarson J Bodin L Tysk C Lindberg E Järnerot G. Inflammatory bowel disease in a Swedish twin cohort: a long-term follow-up of concordance and clinical characteristics. Gastroenterology. (2003) 124:1767–73. doi: 10.1016/s0016-5085(03)00385-8, 12806610

[53] Milajerdi A Ebrahimi-Daryani N Dieleman LA Larijani B Esmaillzadeh A. Association of dietary fiber, fruit, and vegetable consumption with risk of inflammatory bowel disease: a systematic review and Meta-analysis. Adv Nutr. (2021) 12:735–43. doi: 10.1093/advances/nmaa145, 33186988 PMC8166559

[54] Andersen V Chan S Luben R Khaw KT Olsen A Tjonneland A . Fibre intake and the development of inflammatory bowel disease: a European prospective multi-Centre cohort study (EPIC-IBD). J Crohns Colitis. (2018) 12:129–36. doi: 10.1093/ecco-jcc/jjx136, 29373726 PMC5881771

[55] Ananthakrishnan AN Khalili H Konijeti GG Higuchi LM de Silva P Korzenik JR . A prospective study of long-term intake of dietary fiber and risk of Crohn's disease and ulcerative colitis. Gastroenterology. (2013) 145:970–7. doi: 10.1053/j.gastro.2013.07.050, 23912083 PMC3805714

[56] Hossen I Hua W Ting L Mehmood A Jingyi S Duoxia X . Phytochemicals and inflammatory bowel disease: a review. Crit Rev Food Sci Nutr. (2020) 60:1321–45. doi: 10.1080/10408398.2019.1570913, 30729797

[57] Sender R Fuchs S Milo R. Revised estimates for the number of human and Bacteria cells in the body. PLoS Biol. (2016) 14:e1002533. doi: 10.1371/journal.pbio.1002533, 27541692 PMC4991899

[58] Craig WJ Mangels AR Fresan U Marsh K Miles FL Saunders AV . The safe and effective use of plant-based diets with guidelines for health professionals. Nutrients. (2021) 13:4144. doi: 10.3390/nu13114144, 34836399 PMC8623061

[59] Jansakoo T Sekizawa S Fujimori S Hasegawa T Oshiro K. Benefits of air quality for human health resulting from climate change mitigation through dietary change and food loss prevention policy. Sustain Sci. (2024) 19:1391–407. doi: 10.1007/s11625-024-01490-w

[60] Fry A Littlejohns TJ Sudlow C Doherty N Adamska L Sprosen T . Comparison of sociodemographic and health-related characteristics of UK biobank participants with those of the general population. Am J Epidemiol. (2017) 186:1026–34. doi: 10.1093/aje/kwx246, 28641372 PMC5860371

