# Supplementary material for: Association between long-term ambient air pollution and incident inflammatory bowel disease across levels of adherence to a plant-based diet: a prospective cohort study in the UK Biobank
Source: Front Nutr. 2026 Aug 10;13:1893740. doi: 10.3389/fnut.2026.1893740 (PMC13500258; doi:10.3389/fnut.2026.1893740)
Supplement: Supplementary file 1 [file Supplementary_file_1.DOCX]

***Table S1. Digestive system diseases used for baseline exclusion.***

| **Disease category** | **ICD-10 codes** | **ICD-9 codes** | **Self-reported codes** |
| --- | --- | --- | --- |
| Digestive system diseases | K00–K93 | 520–579 | 1134–1143, 1154–1165, 1190–1191,  1400,  1456–1463, 1474–1475, 1501–1506, 1509–1513, 1578–1582, 1599–1606 |

***Notes:*** *Digestive diseases were identified using ICD-9 and ICD-10 codes from hospital inpatient records and self-reported medical conditions. The earliest diagnosis date across data sources was used to determine pre-existing disease status at baseline.*

***Table S2. Diet component definitions and scoring in the PDI.***

| **Food group** | **Dietary variable** | **Field ID in UK Biobank** | **Serving size (g/mL)** | **Scoring for PDI** |
| --- | --- | --- | --- | --- |
| **Plant foods** | | | | |
| whole grains | Type of sliced bread eaten | 20091 | — | Positive |
|  | Type of baguette eaten | 20092 | — |  |
|  | Type of large bap eaten | 20093 | — |  |
|  | Type of bread roll eaten | 20094 | — |  |
|  | Porridge intake | 100770 | 203.5 |  |
|  | Muesli intake | 100800 | 80 |  |
|  | Oat crunch intake | 100810 | 80 |  |
|  | Bran cereal intake | 100840 | 50 |  |
|  | Whole-wheat cereal intake | 100850 | 44 |  |
|  | Other cereal intake | 100860 | 44 |  |
|  | Sliced bread intake | 100950 | 36 |  |
|  | Baguette intake | 101020 | 95 |  |
|  | Bap intake | 101090 | 90 |  |
|  | Bread roll intake | 101160 | 60 |  |
|  | Crispbread intake | 101250 | 10 |  |
|  | Other bread intake | 101270 | 45 |  |
|  | Cereal bar intake | 102370 | 28 |  |
|  | Wholemeal pasta intake | 102720 | 230 |  |
|  | Brown rice intake | 102740 | 150 |  |
|  | Couscous intake | 102770 | 150 |  |
|  | Other grain intake | 102780 | 157 |  |
| fruits | Stewed fruit intake | 104410 | 140 |  |
|  | Prune intake | 104420 | 60 |  |
|  | Dried fruit intake | 104430 | 60 |  |
|  | Mixed fruit intake | 104440 | 105 |  |
|  | Apple intake | 104450 | 112 |  |
|  | Banana intake | 104460 | 100 |  |
|  | Berry intake | 104470 | 40 |  |
|  | Cherry intake | 104480 | 34 |  |
|  | Grapefruit intake | 104490 | 148 |  |
|  | Grape intake | 104500 | 100 |  |
|  | Mango intake | 104510 | 113 |  |
|  | Melon intake | 104520 | 180 |  |
|  | Orange intake | 104530 | 120 |  |
|  | Satsuma intake | 104540 | 70 |  |
|  | Peach/nectarine intake | 104550 | 130 |  |
|  | Pear intake | 104560 | 160 |  |
|  | Pineapple intake | 104570 | 80 |  |
|  | Plum intake | 104580 | 55 |  |
|  | Other fruit intake | 104590 | 60 |  |
| vegetables | Mixed vegetable intake | 104060 | 90 |  |
|  | Vegetable pieces intake | 104070 | 90 |  |
|  | Coleslaw intake | 104080 | 120 |  |
|  | Side salad intake | 104090 | 66 |  |
|  | Beetroot intake | 104130 | 48 |  |
|  | Broccoli intake | 104140 | 80 |  |
|  | Butternut squash intake | 104150 | 130 |  |
|  | Cabbage/kale intake | 104160 | 90 |  |
|  | Carrot intake | 104170 | 60 |  |
|  | Cauliflower intake | 104180 | 90 |  |
|  | Celery intake | 104190 | 30 |  |
|  | Cucumber intake | 104210 | 60 |  |
|  | Garlic intake | 104220 | 5 |  |
|  | Leek intake | 104230 | 80 |  |
|  | Lettuce intake | 104240 | 35 |  |
|  | Mushroom intake | 104250 | 44 |  |
|  | Onion intake | 104260 | 60 |  |
|  | Parsnip intake | 104270 | 65 |  |
|  | Sweet pepper intake | 104290 | 133 |  |
|  | Spinach intake | 104300 | 90 |  |
|  | Sprouts intake | 104310 | 90 |  |
|  | Sweetcorn intake | 104320 | 43 |  |
|  | Sweet potato intake | 104330 | 130 |  |
|  | Fresh tomato intake | 104340 | 85 |  |
|  | Tinned tomato intake | 104350 | 135 |  |
|  | Turnip/swede intake | 104360 | 60 |  |
|  | Watercress intake | 104370 | 20 |  |
|  | Other vegetables intake | 104380 | 60 |  |
| nuts | Salted peanuts intake | 102410 | 40 |  |
|  | Unsalted peanuts intake | 102420 | 40 |  |
|  | Salted nuts intake | 102430 | 40 |  |
|  | Unsalted nuts intake | 102440 | 40 |  |
|  | Seeds intake | 102450 | 14 |  |
| legumes, vegetarian protein alternatives | Vegetarian sausages/burgers intake | 103260 | 90 |  |
|  | Tofu intake | 103270 | 90 |  |
|  | Quorn intake | 103280 | 90 |  |
|  | Other vegetarian alternative intake | 103290 | 90 |  |
|  | Baked bean intake | 104000 | 135 |  |
|  | Pulses intake | 104010 | 70 |  |
|  | Broad bean intake | 104110 | 70 |  |
|  | Green bean intake | 104120 | 70 |  |
|  | Pea intake | 104280 | 65 |  |
| tea and coffee | Instant coffee intake | 100250 | 190 |  |
|  | Filtered coffee intake | 100270 | 190 |  |
|  | Cappuccino intake | 100290 | 160 |  |
|  | Latte intake | 100300 | 160 |  |
|  | Espresso intake | 100310 | 30 |  |
|  | Other coffee type | 100330 | 190 |  |
|  | Standard tea intake | 100400 | 190 |  |
|  | Rooibos tea intake | 100410 | 190 |  |
|  | Green tea intake | 100420 | 190 |  |
|  | Herbal tea intake | 100430 | 190 |  |
|  | Other tea intake | 100440 | 190 |  |
| refined grains | Type of sliced bread eaten | 20091 | — |  |
|  | Type of baguette eaten | 20092 | — |  |
|  | Type of large bap eaten | 20093 | — |  |
|  | Type of bread roll eaten | 20094 | — |  |
|  | Sweetened cereal intake | 100820 | 38 |  |
|  | Plain cereal intake | 100830 | 30 |  |
|  | Sliced bread intake | 100950 | 36 |  |
|  | Baguette intake | 101020 | 95 |  |
|  | Bap intake | 101090 | 90 |  |
|  | Bread roll intake | 101160 | 60 |  |
|  | Naan bread intake | 101230 | 160 |  |
|  | Garlic bread intake | 101240 | 20 |  |
|  | Pancake intake | 102010 | 110 |  |
|  | Scotch pancake intake | 102020 | 41 |  |
|  | Croissant intake | 102050 | 60 |  |
|  | Scone intake | 102070 | 48 |  |
|  | Savoury biscuits intake | 102470 | 40 |  |
|  | Cheesy biscuits intake | 102480 | 40 |  |
|  | Other savoury snack intake | 102500 | 40 |  |
|  | White pasta intake | 102710 | 230 |  |
|  | White rice intake | 102730 | 150 |  |
|  | Snackpot intake | 102760 | 280 |  |
| potatoes | Fried potatoes intake | 104020 | 180 |  |
|  | Boiled/baked potatoes intake | 104030 | 175 |  |
|  | Mashed potato intake | 104050 | 180 |  |
|  | Crisp intake | 102460 | 40 |  |
| sugary drinks | Low calorie drink intake | 100160 | 330 |  |
|  | Fizzy drink intake | 100170 | 330 |  |
|  | Squash intake | 100180 | 250 |  |
| fruit juices | Orange juice intake | 100190 | 250 |  |
|  | Grapefruit juice intake | 100200 | 250 |  |
|  | Pure fruit/vegetable juice intake | 100210 | 250 |  |
|  | Fruit smoothie intake | 100220 | 260 |  |
| sweets and desserts | Double crust pastry intake | 101970 | 60 |  |
|  | Single crust pastry intake | 101980 | 30 |  |
|  | Crumble intake | 101990 | 70 |  |
|  | Yorkshire pudding intake | 102030 | 25 |  |
|  | Danish pastry intake | 102060 | 110 |  |
|  | Fruitcake intake | 102180 | 70 |  |
|  | Cake intake | 102190 | 60 |  |
|  | Doughnut intake | 102200 | 60 |  |
|  | Sponge pudding intake | 102210 | 120 |  |
|  | Other dessert intake | 102230 | 60 |  |
|  | Chocolate bar intake | 102260 | 50 |  |
|  | White chocolate intake | 102270 | 50 |  |
|  | Milk chocolate intake | 102280 | 50 |  |
|  | Dark chocolate intake | 102290 | 50 |  |
|  | Chocolate-covered raisin intake | 102300 | 25 |  |
|  | Chocolate sweet intake | 102310 | 36 |  |
|  | Diet sweets intake | 102320 | 18 |  |
|  | Chocolate-covered biscuits intake | 102340 | 24 |  |
|  | Chocolate biscuits intake | 102350 | 17 |  |
|  | Sweet biscuits intake | 102360 | 17 |  |
|  | Other sweets intake | 102380 | 40 |  |
| **Animal foods** | | | | |
| animal fat | Butter/margarine on bread/crackers | 101300 | — | Negative |
|  | Butter/margarine added to potatoes | 104040 | — |  |
| dairy | Dairy smoothie intake | 100230 | 250 |  |
|  | Milk intake | 100520 | 250 |  |
|  | Flavoured milk intake | 100530 | 250 |  |
|  | Yogurt intake | 102090 | 125 |  |
|  | Ice-cream intake | 102120 | 120 |  |
|  | Low fat hard cheese intake | 102810 | 40 |  |
|  | Hard cheese intake | 102820 | 40 |  |
|  | Soft cheese intake | 102830 | 40 |  |
|  | Blue cheese intake | 102840 | 35 |  |
|  | Low fat cheese spread intake | 102850 | 15 |  |
|  | Cheese spread intake | 102860 | 15 |  |
|  | Cottage cheese intake | 102870 | 60 |  |
|  | Feta intake | 102880 | 40 |  |
|  | Mozzarella intake | 102890 | 40 |  |
|  | Goat's cheese intake | 102900 | 40 |  |
|  | Other cheese intake | 102910 | 40 |  |
|  | Cheesecake intake | 102220 | 110 |  |
|  | Milk-based pudding intake | 102140 | 200 |  |
|  | Other milk-based pudding intake | 102150 | 60 |  |
| eggs | Whole egg intake | 102940 | 50 |  |
|  | Omelette intake | 102950 | 120 |  |
|  | Eggs in sandwiches intake | 102960 | 120 |  |
|  | Scotch egg intake | 102970 | 120 |  |
|  | Other egg intake | 102980 | 75 |  |
| fish or seafood | Tinned tuna intake | 103150 | 92 |  |
|  | Oily fish intake | 103160 | 100 |  |
|  | Breaded fish intake | 103170 | 100 |  |
|  | Battered fish intake | 103180 | 150 |  |
|  | White fish intake | 103190 | 120 |  |
|  | Prawns intake | 103200 | 60 |  |
|  | Lobster/crab intake | 103210 | 85 |  |
|  | Shellfish intake | 103220 | 40 |  |
|  | Other fish intake | 103230 | 100 |  |
| meat | Sausage intake | 103010 | 30 |  |
|  | Beef intake | 103020 | 120 |  |
|  | Pork intake | 103030 | 120 |  |
|  | Lamb intake | 103040 | 120 |  |
|  | Crumbed or deep-fried poultry intake | 103050 | 100 |  |
|  | Poultry intake | 103060 | 130 |  |
|  | Bacon intake | 103070 | 46 |  |
|  | Ham intake | 103080 | 23 |  |
|  | Liver intake | 103090 | 70 |  |
|  | Other meat intake | 103100 | 100 |  |
| miscellaneous animal-based foods | Pizza intake | 102000 | 150 |  |
|  | Indian snacks intake | 102040 | 40 |  |

***TableS3. Definitions and UK Biobank coding of medications.***

| **Category** | **Drug** | **Biobank Code** |
| --- | --- | --- |
| PPIs | Omeprazole | 1140865634 |
|  | Lansoprazole | 1140864752 |
|  | Esomeprazole | 1141177526 |
|  | Rabeprazole | 1141168584 |
|  | Pantoprazole | 1140929012 |
| NSAIDs | Aceclofenac | 1140925806 |
|  | Aspirin | 1140868226 |
|  | Celecoxib | 1141176662 |
|  | Dexketoprofen | 1141164746 |
|  | Diclofenac Sodium | 1140878036 |
|  | Etodolac | 1140871188 |
|  | Etoricoxib | 1141180140 |
|  | Fenoprofen | 1140871226 |
|  | Ibuprofen | 1140871310 |
|  | Ketoprofen | 1140871506 |
|  | Mefenamic Acid | 1140871542 |
|  | Meloxicam | 1140926732 |
|  | Nabumetone | 1140875336 |
|  | Naproxen | 1140871462 |
|  | Piroxicam | 1140871666 |
|  | Sulindac | 1140871604 |
|  | Tenoxicam | 1140875346 |
|  | Tiaprofenic Acid | 1140871614 |

***TableS4.*** ***Independent associations between PDI and AP with IBD risk in sensitivity analyses.***

| **Variables** | **HR (95% CI)** | | | |
| --- | --- | --- | --- | --- |
|  | **Sensitivity analysis 1** | **Sensitivity analysis 2** | **Sensitivity analysis 3** | **Sensitivity analysis 4** |
| ***IBD：AP (per IQR change)*** |  |  |  |  |
| PM_2.5_ | 1.341 (1.171 - 1.536) | 1.353 (1.156 - 1.585) | 1.307 (1.155 - 1.48) | 1.296 (1.145, 1.465) |
| PM_10_ | 1.251(1.122 - 1.395) | 1.249 (1.104 - 1.413) | 1.184 (1.025 - 1.368) | 1.222 (1.105, 1.350) |
| NO_X_ | 1.178(1.078 - 1.287) | 1.185 (1.068 - 1.315) | 1.140 (1.013 - 1.282) | 1.149 (1.060, 1.245) |
| NO_2_ | 1.201(1.09 - 1.324) | 1.205 (1.075 - 1.351) | 1.158 (1.017 - 1.318) | 1.172 (1.072, 1.281) |
| SO_2_ | 1.129(1.042 - 1.223) | 1.244 (1.108 - 1.396) | 1.124 (1.018 - 1.241) | 1.116 (1.043, 1.194) |
| Benzene | 1.249(1.131 - 1.38) | 1.271 (1.136 - 1.422) | 1.261 (1.104 - 1.441) | 1.224 (1.116, 1.343) |
| ***CD：AP (******per IQR change)*** |  |  |  |  |
| PM_2.5_ | 1.324(1.058 - 1.657) | 1.307 (1.011 - 1.69) | 1.265 (1.027 - 1.559) | 1.255 (1.019, 1.544) |
| PM_10_ | 1.205(1.007 - 1.441) | 1.182 (0.967 - 1.444) | 1.092 (0.849 - 1.403) | 1.168 (0.987, 1.381) |
| NO_X_ | 1.143(0.988 - 1.324) | 1.108 (0.931 - 1.318) | 1.165 (0.955 - 1.422) | 1.104 (0.963, 1.267) |
| NO_2_ | 1.155(0.983 - 1.358) | 1.114 (0.922 - 1.345) | 1.161 (0.929 - 1.451) | 1.119 (0.962, 1.301) |
| SO_2_ | 1.232(1.093 - 1.387) | 1.350 (1.127 - 1.617) | 1.180 (1.002 - 1.39) | 1.179 (1.061, 1.310) |
| Benzene | 1.253(1.063 - 1.477) | 1.265 (1.055 - 1.518) | 1.226 (0.97 - 1.549) | 1.197 (1.024, 1.399) |
| ***UC：AP (per IQR change)*** |  |  |  |  |
| PM_2.5_ | 1.400(1.191 - 1.646) | 1.462 (1.211 - 1.765) | 1.374 (1.186 - 1.592) | 1.361 (1.176, 1.576) |
| PM_10_ | 1.306(1.147 - 1.487) | 1.339 (1.155 - 1.551) | 1.247 (1.05 - 1.482) | 1.278 (1.135, 1.439) |
| NO_X_ | 1.213(1.092 - 1.346) | 1.258 (1.115 - 1.419) | 1.146 (0.994 - 1.321) | 1.188 (1.081, 1.307) |
| NO_2_ | 1.249(1.113 - 1.401) | 1.296 (1.134 - 1.481) | 1.180 (1.01 - 1.378) | 1.221 (1.099, 1.356) |
| SO_2_ | 1.073(0.971 - 1.186) | 1.208 (1.051 - 1.387) | 1.092 (0.966 - 1.234) | 1.084 (0.997, 1.178) |
| Benzene | 1.276(1.134 - 1.435) | 1.324 (1.16 - 1.512) | 1.306 (1.115 - 1.53) | 1.266 (1.134, 1.412) |

***Sensitivity analysis 1:*** *excluding participants diagnosed with IBD within the first two years of follow-up.*

***Sensitivity analysis 2:*** *excluding participants diagnosed with IBD within the first five years of follow-up.*

***Sensitivity analysis 3:*** *additionally adjusting for the use of proton pump inhibitors (PPIs) or non-steroidal anti-inflammatory drugs (NSAIDs), as well as vitamin, mineral, or dietary supplement use.*

***Sensitivity analysis 4:*** *performing multiple imputation by chained equations for covariates with missing values.*

# *Table S5. Two-pollutant Cox models in sensitivity analyses for IBD, CD and UC*

| **Exposure** | **IBD HR (95% CI)** | **CD HR (95% CI)** | **UC HR (95% CI)** |
| --- | --- | --- | --- |
| PM_2.5_+SO_2_ | 1.237(1.033 - 1.480) | 1.151(0.840 - 1.576) | 1.307(1.055 - 1.619) |
| PM_10_+SO_2_ | 1.164(1.007 - 1.346) | 1.066(0.827 - 1.375) | 1.231(1.035 - 1.464) |
| NO_X_+SO_2_ | 1.109(0.982 - 1.253) | 1.121(0.911 - 1.380) | 1.124(0.971 - 1.302) |
| NO_2_+SO_2_ | 1.122(0.980 - 1.284) | 1.107(0.876 - 1.397) | 1.155(0.983 - 1.357) |
| Benzene+SO_2_ | 1.225(1.065 - 1.408) | 1.161(0.909 - 1.483) | 1.288(1.091 - 1.521) |

***Abbreviations:*** *SO_2_ was selected as the co-pollutant in two-pollutant models because it exhibited relatively weak correlations with other air pollutants (Figure S1). IBD, Inflammatory Bowel Disease; UC, Ulcerative Colitis; CD, Crohn’s Disease; PM_2.5_, particulate matter with aerodynamic diameter <2.5 μm; PM₁₀, particulate matter with aerodynamic diameter <10 μm; NO₂, nitrogen dioxide; NOx, nitrogen oxides; SO₂, sulfur dioxide.*

***Figure S1: Spearman correlation matrix among ambient air pollutants.*** *PM_2.5_, particulate matter with aerodynamic diameter <2.5 μm; PM₁₀, particulate matter with aerodynamic diameter <10 μm; NO₂, nitrogen dioxide; NOx, nitrogen oxides; SO₂, sulfur dioxide.*

***Figure S2: Exposure–response relationships between*** ***ambient air pollutants and the risk of Crohn’s disease.*** *Models were adjusted for age, sex, ethnicity, BMI, employment status, education, household income, physical activity, smoking status, and alcohol consumption. Panels A–F correspond to the following pollutants: A: PM_2.5_, particulate matter with aerodynamic diameter <2.5 μm, B: PM₁₀, particulate matter with aerodynamic diameter <10 μm, C: NOx, nitrogen oxides, D: NO₂, nitrogen dioxide, E: SO₂, sulfur dioxide, and F: benzene.*

***Figure S3: Exposure–response relationships between ambient air pollutants and the risk of ulcerative colitis.*** *Models were adjusted for age, sex, ethnicity, BMI, employment status, education, household income, physical activity, smoking status, and alcohol consumption. Panels A–F correspond to the following pollutants: A: PM_2.5_, particulate matter with aerodynamic diameter <2.5 μm, B: PM₁₀, particulate matter with aerodynamic diameter <10 μm, C: NOx, nitrogen oxides, D: NO₂, nitrogen dioxide, E: SO₂, sulfur dioxide, and F: benzene.*
